# *Commiphora myrrha* Resin Alcoholic Extract Ameliorates High Fat Diet Induced Obesity via Regulation of UCP1 and Adiponectin Proteins Expression in Rats

**DOI:** 10.3390/nu12030803

**Published:** 2020-03-18

**Authors:** Sahar H. Orabi, Eman SH. Al-Sabbagh, Hanem K. Khalifa, Mostafa Abd El-Gaber Mohamed, Moustafa Elhamouly, Shaban M. Gad-Allah, Mohamed M. Abdel-Daim, Mabrouk A. Abd Eldaim

**Affiliations:** 1 Department of Biochemistry and Chemistry of Nutrition, Faculty of Veterinary Medicine, University of Sadat City, Sadat City 32958, Egypt; saher977@yahoo.com (S.H.O.); mnsihab@yahoo.com (E.S.A.-S.); hanemkamalbasuni@yahoo.com (H.K.K.); 2 Department of Pathology, Faculty of Veterinary Medicine, Menoufia University, Shebeen Elkom 32511, Egypt; mostafa.abdelgaber@vet.usc.edu.eg; 3 Department of Histology and Cytology, Faculty of Veterinary Medicine, University of Sadat City, Sadat City 32958, Egypt; mostafa.elhamoli@vet.usc.edu.eg; 4 Department of Surgery, Faculty of Veterinary Medicine, University of Sadat City, Sadat City 32958, Egypt; Shabangadallah@vet.usc.edu.eg; 5 Department of Zoology, College of Science, King Saud University, P.O. Box 2455, Riyadh 11451, Saudi Arabia; 6 Pharmacology Department, Faculty of Veterinary Medicine, Suez Canal University, Ismailia 41522, Egypt; 7 Department of Biochemistry and Chemistry of Nutrition, Faculty of Veterinary Medicine, Menoufia University, Shebeen Elkom 32511, Egypt

**Keywords:** *Commiphora myrrha*, obesity, leptin, adiponectin, UCP1

## Abstract

This study was performed to evaluate anti-obesity potential of *Commiphora myrrha* resin ethanolic extract (CME) with the respect to expression of leptin, adiponectin and uncoupling protein 1 (UCP1) in rats. Control rats fed basal diet. Second group fed basal diet and administered CME (500 mg/kg bw) orally for 14 weeks. Third group fed high fat diet (HFD) for 14 weeks. Fourth group fed HFD and administered CME as second group. Fifth group fed HFD for 8 weeks then fed basal diet and administered CME as third group for another 6 weeks. Phytochemical analysis of CME identified the presence of germacrene B, 1,4-benzoquinone, benzofuran, hexadecanoic acid, 9,12-octadecnoic acid methyl ester, reynosin, 11, 14-eicosadienoic acid, isochiapin B, bisabolene epixod, elemene and 1-heptatriacotanol. High fat diet significantly increased food intake, body weight, hyperglycemia, serum levels of total cholesterol, triacylglycerol, low density lipoprotein and ketone bodies, AST and AST activities, concentration of malondialdehyde and histopathological changes in hepatic tissues. However, it significantly reduced serum levels of high density lipoprotein, leptin and adiponectin, activity of hepatic glutathione reductase (GR) and brown adipose tissue UCP1 protein expression. In contrast, CME ameliorated HFD increased body weight, hyperglycemia, dyslipidemia, ketonemia, hepatic tissues lipid peroxidation, restored hepatic tissue architecture and enhanced protein expression of leptin, adiponectin and UCP1 and activity of hepatic GR. This study indicated that CME ameliorated HFD induced hyperglycemia and dyslipidemia through normalization of HFD reduced leptin, adiponectin and UCP1 proteins production and antioxidant activity.

## 1. Introduction

Obesity is a serious problem that affects human health leading to several diseases including, diabetes mellitus, hypertension, and cardiovascular diseases [1]. The World Health Organization (WHO) has recognized that the high blood cholesterol is the main cause of cardiovascular diseases worldwide leading to death of about 4.4 million people each year. The main character of hyperlipidemia is the elevated levels of serum low density lipoprotein, very low density lipoprotein, and reduced level of high density lipoprotein [2]. Consumption of human and rodents’ high fat diet induces obesity and hyperlipidemia associated with altered plasma and tissues cholesterol and triacylglycerol levels that promote the risk of coronary heart disease, fatty liver, and carcinogenesis [3]. Liver plays vital roles in lipid metabolism and disturbance in lipid metabolism due to high access of fat to the liver, lower mobilization of fat from liver or defect in the structure and/or function of lipoproteins leads accumulation of fat in the liver, which subsequently induced hepatomegaly, changes in the shape and colors of the liver. It is well documented that HFD induced liver disturbances including hepatomegaly [4,5]. 

The development and progression of obesity, dyslipidemia and diabetes mellitus type 2 are regulated by some proteins and cytokines such as leptin, adiponectin and uncoupling protein 1. Leptin is an adipocyte derived hormone secreted from adipocyte and plays an important role in amelioration of complications and pathogenesis of obesity [6,7] as it acts on the hypothalamus to control appetite, food intake, energy metabolism, and sympathetic nervous system outflow [7,8]. Adiponectin is an adipocyte derived hormone, whose plasma concentrations are decreased in obese and type 2 diabetes mellitus subjects. It has anti-atherogenic and anti-inflammatory properties [9]. Over nutrition leads to hypoadiponectinemia and increased TNF-α that enhanced insulin resistance [10]. Adiponectin improves diabetic mice through enhancement of free fatty acid (FFA) oxidation in muscle, clearance of plasma FFA in HFD [11] and decreases glucose release from hepatocyte [12]. Uncoupling protein 1 (UCP1) is implicated in thermogenesis, energy expenditure, and reduction of oxidative stress that associated with the progress of obesity and diabetes mellitus type 2 (DM2) [13]. In addition, UCP1 has a role in regulation of cold and diet-induced thermogenesis, metabolic and energy balance and reducing the mitochondrial production of reactive oxygen species (ROS) that involved in the pathogenesis and progression of obesity and/or DM2 [14].

Treatment of obesity and dyslipidemia by synthetic chemical drugs causes several adverse side effects; therefore, it is of great interest to look for alternative safe natural agents. Medicinal plants contain various bioactive substances such as phytosterols, diterpenes, triterpenes, and polyphenolic compounds that enable them to prevent and treat many disorders [2,15].

*Commiphora myrrha* (CM) (family Burseraceae) is a small tropical tree that is widely distributed in East Africa, Arabia and India [16]. Myrrh is a resinous exudate obtained from the trunk of CM trees [17]. *Commiphora myrrha* contains many bioactive substances thus; it was used to treat a variety of illnesses such as obesity and lipid disorders [18]. In addition, it has anti-hyperglycemic, antioxidant [19], hepatoprotective [20], analgesic, anti-inflammatory [21], hypolipidemic [22], and cholesterol lowering activities through inhibiting LDL oxidation [23]. Studies concerning the anti-obesity activity of CM extract still scarce to the best of our knowledge. Therefore, this study was performed to evaluate the preventive and curative effects of CM resin alcoholic extract against HFD induced obesity and dyslipidemia with the respect to its impact on the expression of obesity development related cytokines, leptin, adiponectin and UCP1 in rats.

## 2. Materials and Methods

### 2.1. Materials

#### 2.1.1. *Commiphora myrrha*

*Commiphora myrrha* resin (yellowish crystals), family Burseraceae, was purchased from commercial herpes shop at Alexandria, Egypt. *Commiphora myrrha* resin was identified and authenticated at the department of Biochemistry and Chemistry of Nutrition, Faculty of Veterinary Medicine, University of Sadat City. It was washed, dried and grinds into a fine powder.

#### 2.1.2. Animals

Fifty adult male Wister albino rats of 6 weeks old and weighting 100–120 g were used in this experiment. Animals were housed in wide plastic cages at 18–22 °C temperature and 30–60% humidity with natural ventilation and 12 h light/dark cycle. Rats were provided with balanced ration and tap water ad libitum and kept for two weeks for acclimatization before the beginning of the experiment. The experimental design was approved by the Research Ethical Committee of the Faculty of Veterinary Medicine, University of Sadat City with approval number, Vusc-003-2-20.

#### 2.1.3. Diagnostic Kits

Diagnostic Kits used for analysis of serum levels of glucose, triacylglycerol (TA), total cholesterol (TC), high density lipoprotein (HDL) and ketone bodies, activities of serum alanine aminotransferase (ALT) and aspartate aminotransferase (AST), the concentration of malondialdehyde (MDA) and the activity of glutathione reductase (GR) were purchased from Bio-diagnostic Company, Cairo, Egypt. Diagnostic kits used for Enzyme Linked Immune Sorbent Assay (ELISA) for determination of serum leptin and adiponectin levels were purchased from Nova Chemicals Company, Cairo, Egypt. Diagnostic kits for analysis of UCP-1 protein expression in brown adipose tissue were obtained from Santa Cruz Biotechnology, Inc., Dallas, TX, USA.

### 2.2. Methods

#### 2.2.1. Preparation of Diet

##### Basal Diet

The basal ration was purchased from Al Wady Company, Cairo, Egypt. A basal diet composed of yellow corn, soybean seeds, soybean oil, limestones, monocalcium phosphate, sodium chloride, sodium bicarbonate, lecithin and mixture of minerals and vitamins (Table 1). 

##### High-Fat Diet (HFD)

High fat diet was prepared according to [4]. It composed basal diet in addition of 7% beef tallow, 8.3% yolk, 18.7% sucrose, and 66% standard diet (Table 2).

#### 2.2.2. Preparation of *Commiphora myrrha* Resin Ethanolic Extract

After grinding of the resin into a fine powder, 800 g of the powder was soaked in 90% ethanol and kept in a fridge with intermittent shaking for 72 h. The extract was filtered by using Whatman filter paper No 1. Then the extract was concentrated under reduced pressure by rotary evaporator at 50 °C [24]. Finally, the extract was kept in a refrigerator till usage. 

#### 2.2.3. Gas Chromatography–Mass Spectrometry (GC-MS) Analysis of *Commiphora myrrha* Resin Extract

The phytochemical analysis of *Commiphora myrrha* resin ethanolic extract was performed by using Trace GC1300-TSQ mass spectrometer (Thermo Scientific, Austin, TX, USA) with a direct capillary column TG–5MS (30 m × 0.25 mm × 0.25 µm film thickness). The temperature of column oven was firstly held at 50 °C and increased by 5 °C/min till reach 250 °C and hold at that temperature for 2 min finally the temperature was increased by 25 °C/min to reach 300 °C and hold at it for 2 min. The temperatures of the injector and MS transfer line were kept at 250, 260 °C respectively. Helium was used as a carrier gas at a constant flow rate of 1 mL/min. The solvent delay was 3 min and diluted samples of 1 µL were injected automatically using Autosampler AS1300 coupled with GC in the split mode. EI mass spectra were collected at 70 eV ionization voltages over the range of m/z 50–650 in full scan mode. The temperature of ion source was set at 250 °C. The components were identified by comparison of their retention times and mass spectra with those of WILEY 09 and NIST 11 mass spectral database.

#### 2.2.4. Experimental Design

A total of 50 male albino rats were randomly distributed into five groups, 10 rats each.

- **Control group:** Rats were fed a basal diet for 14 weeks.
- **Second group (CME group):** Rats were fed a basal diet and administrated *Commiphora myrrha* resin ethanolic extract (CME) orally at a dose of 500 mg/kg body weight for 14 weeks [21].
- **Third group (HFD group):** Rats were fed HFD for 14 weeks [4].
- **Fourth group (HFD & CME group):** Rats were fed HFD and administered CME orally at a dose of 500 mg/kg for 14 weeks.
- **Fifth group (HFD then basal diet and CME):** Rats were fed HFD for 8 weeks then fed a basal diet and administered CME orally at a dose of 500 mg/kg body weight for another 6 weeks.

#### 2.2.5. Sampling

Blood samples were collected after 8 and 14 weeks from the beginning of the experiment. Rats were anesthetized by using phenobarbital at dose 45 mg/kg bw i.p and blood samples were withdrawn from medial canthus of the eyes with heparinized capillary tube and were left at room temperature for clotting. Then Sera samples were collected and kept at −80 °C till usage for biochemical and cytokines investigations. 

##### Tissue Sampling

By the end of the experiment rats were anesthetized and scarified by decapitation and livers were removed and weighted then washed in saline. Brown adipose and specimens of liver were washed in saline and then kept immediately in 10% formalin for histopathological and immunohistochemical investigations. 

#### 2.2.6. Measurements

Food intake was recorded every day. Rats were weighed every week. At the end of the experiment livers of rats were weighed.

#### 2.2.7. Biochemical Analysis of Serum Metabolites and Livers Lipid Peroxidation and Antioxidant Activity

Blood glucose level was analyzed by using enzymatic colorimetric methods as described by [25] where glucose oxidase enzyme oxidized glucose to gluconic acid and the formed hydrogen peroxide is estimated by a colorimetric oxygen acceptor’ phenol, 4-aminophenazone (4-AP) in the presence of peroxidase enzyme at wavelength 505 nm. Serum total cholesterol (TC) levels were measured colorimetrically through using cholesterol esterase, cholesterol oxidase and indicator reaction with peroxidase, 4-aminophenazone and phenol according to [26]. Serum levels of triacylglycerides (TG) were estimated calorimetrically according to [27]. Serum levels of high density lipoprotein cholesterol (HDL) were determined according to [28]. High density lipoprotein cholesterol was isolated by precipitation of chylomicrons, very low-density lipoprotein cholesterol and low-density lipoprotein cholesterol (LDL-C) by addition of phosphotungstic acid and magnesium ions to samples. Then HDL levels were determined in the clear supernatant at wavelength 505 nm. Low density lipoproteins (LDL) cholesterol and very low density lipoprotein (VLDL) were calculated according to [19]. Serum levels of both acetoacetic acid and beta hydroxybutyric acid (ketone bodies) were analyzed according to [29] by using a ketone body assay kit where the reaction was catalyzed by 3-hydroxybutyrate dehydrogenase, the absorbance was measured at wavelength 340 nm and the standard curve was used to quantify the concentrations acetoacetic acid and beta hydroxybutyric acid. The total concentration of ketone bodies equals the sum of the concentrations for both acetoacetic acid and beta hydroxybutyric acid. Activities of serum ALT and AST were determined according to [30] where ALT catalyzes the transfer of amino group of alanine to α ketoglutarate while AST catalyzes the transfer of amino group of aspartate to α ketoglutarate. The transaminase activity is proportional to the amount of pyruvate formed over a definite period of time and is measured by a reaction with 2.4-dinitrophenylhydrazine (DNPH) in alkaline solution forming colored substance that can be measured spectrophotometrically at wavelength 505 nm. The concentrations of malondialdehyde (MDA) in hepatic tissues were measured according to [31]. Thiobarbituric acid (TBA) reacts with malondialdehyde (MDA) in acidic medium at a temperature of 95 °C for 30 min to produce thiobarbituric acid reactive product. The absorbance of the resultant pink product measured spectrophotometrically at wavelength 534 nm. The activity of hepatic tissues glutathione reductase (GR) was determined according to [32]. Glutathione reductase catalyzes the reduction of oxidized glutathione (GSSG) into reduced glutathione (GSH) in the presence of NADPH, which is oxidized to NAD. The decrease in absorbance is measured at wavelength 340 nm. 

#### 2.2.8. Assessment of Serum Levels of Leptin and Adiponectin

Serum levels of leptin and adiponectin were analyzed according to [33] by using ELISA assay kits for determination of serum levels of leptin and adiponectin (cat. numbers 201-11-0456 and 201-11-0759, respectively, Shanghai Sunred Biological Technology Co., Ltd., Shanghai, China) according to manufacturer instruction. The optical density (OD) was measured at wavelength 450 nm by using Stat Fax^®^ 4200 Micro Plate Reader, Awareness Technology, Palm City, FL, USA. The concentrations of leptin or adiponectin in serum samples were calculated by using the standard curve linear regression equation. The sensitivity of the assay to detect the leptin was 7.054 pg/mL and the assay range was 7.5–2000 pg/mL. While the sensitivity of the assay to detect the adiponectin was 0.228 mg/L and the assay range was 0.5–60 mg/L. The specificity for intra-Assay: CV<9% and inter-Assay: CV<11% where CV (%) = SD/mean × 100.

#### 2.2.9. Histopathological Studies

Liver tissue samples were fixed in 10% neutral buffered formalin. Fixed samples were processed and stained with hematoxylin and eosin (H&E) according to [34].

#### 2.2.10. Immunohistochemistry for Detection of UCP-1 Protein

Uncoupling protein 1 protein expression in brown adipose tissue was detected immunohistochemically according to [35]. Tissues sections were dewaxed hydrated, immersed in EDTA solution (antigen retrieval solution) at pH 8, treated with 0.3% hydrogen peroxide and protein block and incubated with UCP1 primary antibody (A-6-sc-518024, Santa Cruz biotechnology, Inc.; 1:50 dilution). Then slides were rinsed with PBS, incubated with anti-mouse IgG secondary antibodies (EnVision + System HRP; Dako, Fargo, ND, USA) for 30 min at room temperature, visualized with di-aminobenzidine commercial kits (Liquid DAB+Substrate Chromogen System; Dako), and finally counterstained with Mayer’s haematoxylin. For negative control, the primary antibody was replaced by normal mouse serum. The labelling index of UCP1 were expressed as the percentage of positive area cells in about 8 high power fields using Image J analysis software (NIH, Bethesda, MD, USA).

#### 2.2.11. Statistical Analysis

Results were expressed as mean ± SE. Data were analyzed by ANOVA followed by Duncan’s post hoc test for significant difference (*p* < 0.05) according to [36]. The statistical analysis was carried out using SPSS (Statistical Package for Social Sciences) Version 16 released in 2007.

## 3. Results

### 3.1. The Phytochemical Components of Commiphora myrrha Resin Ethanolic Extract

**Figure 1:**
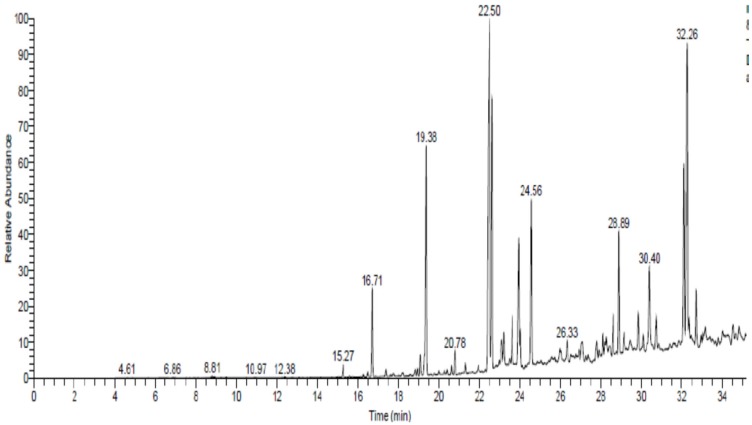
The spectrum of identified compounds from *Commiphora myrrha* resin ethanolic extract by using Trace GC1300-TSQ mass. *Commiphora myrrha* resin ethanolic extract were prepared and phytochemically analyzed using Trace GC1300-TSQ mass.

Phytochemical analysis of *Commiphora myrrha* resin ethanolic extract by using GC1300 mass spectrometer indicated the presence of germacrene B, 1,4-benzoquinone, benzofuran, hexadecanoic acid, 9,12-octadecnoic acid methyl ester, reynosin, 11, 14-eicosadienoic acid, isochiapin B, bisabolene epixod, elemene and 1-heptatriacotanol (Figure 1 and Table 3).

### 3.2. Commiphora myrrha Resin Extract Reduced HFD Increased Food Intake and Body Weight in Rats

**Figure 2:**
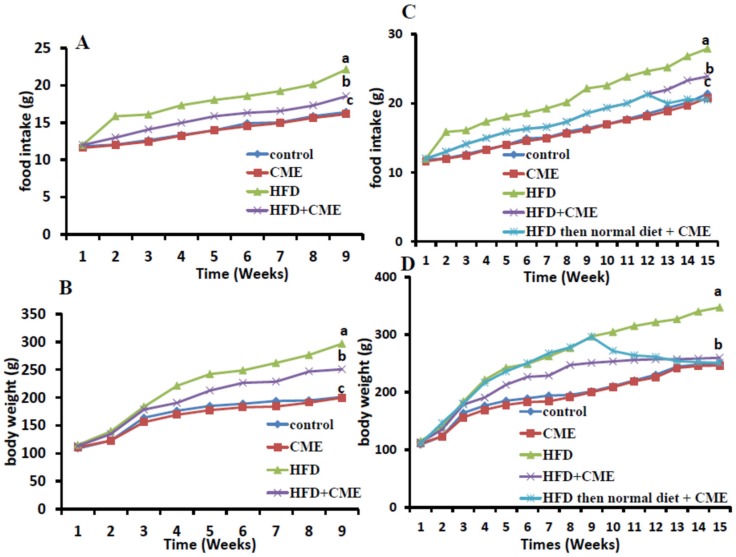
Effect of high fat diet (HFD) and/or *Commiphora myrrha* resin extract (CME) on food intake and body weights. Rats were fed either basal (normal control group) diet; basal diet and administered CME (500 mg/kg bw) for 14 weeks; HFD; HFD and administered CME (500 mg/kg bw) for 14 weeks or HFD for 8 weeks then basal diet and administered CME (500 mg/kg bw) for another 6 weeks. Daily food intake after 8 weeks (**A**) and after 14 weeks (**C**) and weekly live body weight of rats after 8 weeks (**B**) and after 14 weeks of the experiment (**D**) were recorded. Values presented are the mean ± SE. Different letters means significant difference (*p* < 0.05) from the other groups.

Figure 2 showed the effect of HFD and/or CME on food intake and body weight of rats after 8 weeks and at the end of the experiment. Feeding of rat HFD significantly increased (*p* < 0.05) food intake and body weight compared with the normal control group that fed basal diet after 8 weeks and at the end of the experiment (Figure 2A,C). Administration of rats fed HFD with CME (HFD+ CME group) significantly reduced food intake and body weight compared with those fed HFD (HFD group) after 8 and 14 weeks (Figure 2B,D). Supplementation of rats that fed HFD diet from the beginning till 8th week of the experiment then feed on basal diet till the end of the experiment with CME from the beginning of 9th week till the end of the experiment (HFD then normal diet + CME group) reduced food intake and body weights gradually till normalized it by the end of the experiment compared with those fed HFD (HFD group) till the end of the experiment (Figure 2B,D). On the other hand, supplementation of rats of the 2nd group that fed basal diet with CME had no significant effect on food intake and body weight compared with those of the normal control rats that fed basal diet (Figure 2A–D).

**Figure 3:**
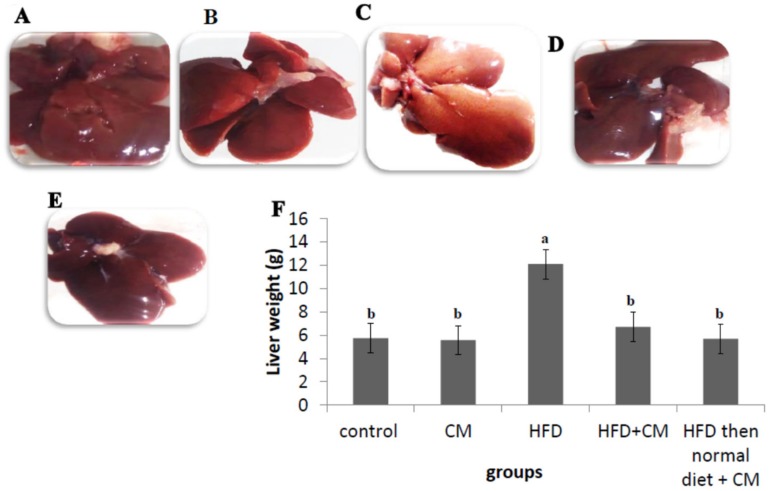
The effect of high fat diet (HFD) and/or *Commiphora myrrha* resin extract (CME) on liver gross appearance and weights. Rats were reared, fed and treated as in Figure 1. Images of the gross appearance of representative livers from the different groups were shown (**A–E**). (**A**,**B**) showed a normal red color of livers of the control and CME rats. (**C**) showed pale yellow color with friable edges of the liver of HFD group. (**D**,**E**) showed normal color (dark red), smooth texture and sharp edges livers of HFD+ CME and HFD then normal diet + CME groups. Livers of each rat were weighed at the end of experiment (**F**). Values presented are the mean ± SE. Columns with different letters means significant difference (*p* < 0.05) from the other groups.

In addition, feeding rats HFD (HFD group) altered the gross appearance of the livers as they were pale yellow color with friable edges (Figure 3C). However, supplementation of rats of HFD+ CME and HFD then normal diet+ CME groups with CME improved livers gross appearance as they had normal color (dark red), smooth texture and sharp edges (Figure 3E,F). On the other hand, livers of the control and CME supplemented rats (control and CME groups) had normal gross appearance (Figure 3A,B). Moreover, feeding rats of the 3rd group with HFD significantly increased livers weights compared to those of the normal control rats fed basal diet. In contrast, supplementation of rats of HFD+ CME and HFD then normal diet+ CME groups with CME normalized livers weights (Figure 3G).

### 3.3. Commiphora myrrha Resin Extract Ameliorated HFD Induced Hyperglycemia and Dyslipidemia in Rats

Feeding of rats of the HFD group with HFD significantly increased (*p* < 0.05) blood glucose level and serum levels of TC, TG, LDL, and VLDL however, it significantly decreased serum levels of HDL compared with control group after 8th weeks of the experiment. On contrast, supplementation of rats of HFD+ CME group with CME significantly reduced (*p* < 0.05) blood glucose level and serum levels of TC, TG, LDL, and VLDL while it significantly increased the serum levels of HDL compared with those of the CME group that fed HFD (Table 4). 

Moreover, feeding rats of HFD group HFD for 14 weeks significantly increased (*p* < 0.05) blood glucose levels, serum levels of TC, TG, LDL, VLDL, ketone bodies and activities of serum ALT, and AST, while it significantly decreased serum levels of HDL compared with the normal control group that fed basal diet. On contrast, Administration of rats of the HFD+ CME group (that HFD for 14 weeks) and those of HFD then normal diet+ CME group (that fed HFD for 8 weeks then fed basal diet until the end of the experiment) with CME significantly reduced (*p* < 0.05) blood glucose levels, serum levels of TC, TG, LDL, VLDL, ketone bodies, and activities of serum AST, and ALT. However, it significantly increased serum levels of HDL compared with rats fed HFD until the end of the experiment (HFD group) (Table 3). *Commiphora myrrha* resin extract itself had no significant effect on blood glucose level and serum levels of TC, TG, LDL, VLDL, and HDL compared with the control group (Table 4 and Table 5). 

### 3.4. Commiphora myrrha Resin Extract Modulates HFD Increased Lipid Peroxidation and Altered Antioxidant Enzyme Activity of Rats

Table 6 showed that HFD significantly increased lipid peroxidation biomarker, MDA, concentration however; it significantly decreased the activity of GR in hepatic tissues compared with the control group. On the contrast, administration of rats of HFD+ CME group and HFD then normal diet+ CME group with CME significantly decreased MDA concentration while increased the activity of GR in hepatic tissues compared with rats fed HFD (HFD group).

### 3.5. Commiphora myrrha Resin Extract Modulated HFD Induced Histopathological Changes in Hepatic Tissues of Rats

**Figure 4:**
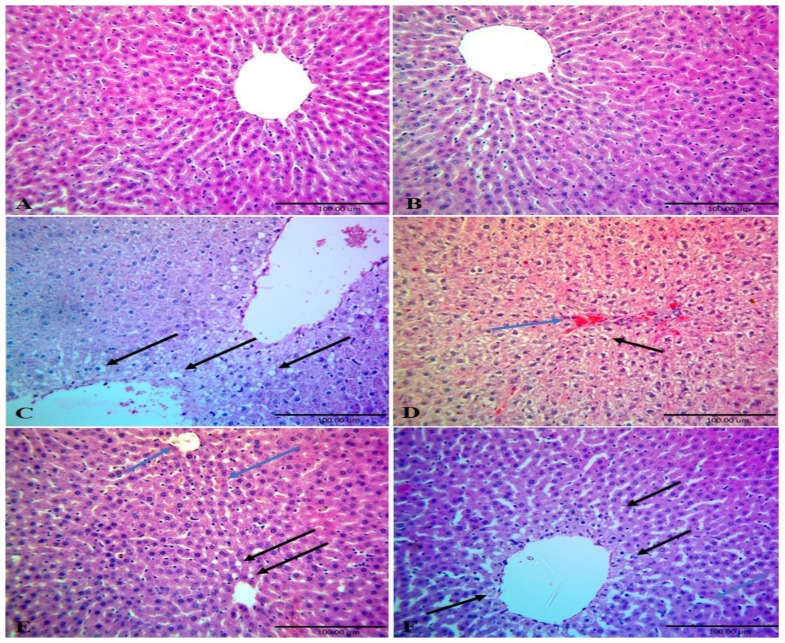
Photomicrograph of liver tissues sections of different groups stained with H&E. Rats were reared, fed and treated as in Figure 1. Livers tissues of normal control group (**A**) CME treated group (**B**) showed normal hepatic tissue. Livers sections of HFD group (**C** and **D**) showed congested blood vessels (blue arrow) with diffuse fatty change of hepatocytes (black arrow). Livers tissues sections of group HFD and CME treated group (**E**) showed slight congestion of central vein and hepatic sinusoids (blue arrow), some hepatocytes showed fatty change (black arrow). Liver tissues sections of HFD then normal diet+ CME group (**F**) showed dilated hepatic sinusoids (blue arrow), hepatocytes showed multiple fat globules appear as clear vacuoles of different size (black arrow) (X40).

Figure 4 illustrated the histopathological changes in the hepatic tissues of the different experimental groups. Hepatic tissue architectures of the normal control and CME administrated groups, respectively were normal (Figure 4A,B). Liver tissue of HFD fed rats (HFD group) showed congested blood vessels (blue arrow) with diffuse fatty change of hepatocytes (Figure 4C,D). Hepatic tissues of rats fed HFD and administered with CME simultaneously for 14 weeks (HFD+ CME group) showed slight congestion of central vein and hepatic sinusoids and some hepatocytes showed fatty change (Figure 4E). Liver tissues sections of rats fed HFD for 8 weeks and then fed basal diet and treated with CME for another 6 weeks showed dilated hepatic sinusoids and hepatocytes showed multiple fat globules appeared as clear vacuoles of different sizes (Figure 4F).

### 3.6. Commiphora myrrha Resin Extract Elevated Serum Levels of Leptin and Adiponectin of HFD Fed Rats

Table 7 showed serum levels of leptin and adiponectin of different experimental groups. HFD significantly reduced (*p* < 0.05) serum levels of both leptin and adiponectin compared with the normal control group fed basal diet. However, administration of rats fed HFD either for 14 weeks (HFD+ CME group) or for 8 weeks and then fed basal diet for another 6 weeks (HFD then normal diet+ CME group) with CME normalized serum levels of leptin and adiponectin compared with those of rats fed HFD only (3rd group) (Table 7). 

### 3.7. Commiphora myrrha Resin Extract Increased UCP1 Protein Expression in Brown Adipose Tissue of Rats Fed HFD

**Figure 5:**
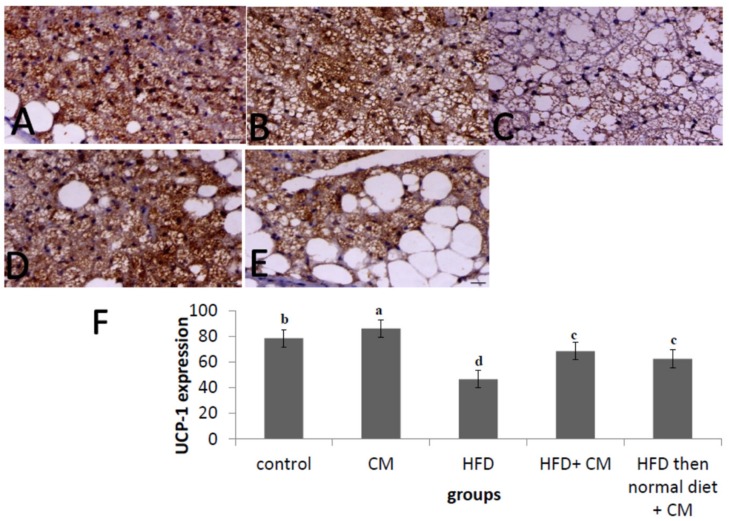
Photomicrograph of brown adipose tissues sections of different groups immunostained with immune reactive UCP1 primary antibody. Rats were reared, fed and treated as Figure 1. Brown adipose tissue of control animal showing marked expression of UCP1 within the cytoplasm of adipocytes, UCP1 IHC, bar = 40 µm, X400 (**A**). Brown adipose tissue of normal animal supplemented with Myrrha showing marked expression of UCP1, UCP1 IHC, bar = 40 µm, X400 (**B**). Brown adipose tissue of HFD-treated animal showing marked decrease the expression of UCP1 within the cytoplasm of adipocytes, UCP1 IHC, bar = 40 µm, X400 (**C**). Brown adipose tissue of HFD + Myrrha-treated animal (14 weeks) showing increase the expression of UCP1, UCP1 IHC, bar = 40 µm, X400 (**D**). Brown adipose tissue of HFD for (8 weeks) then basal diet + Myrrha-treated animal (6 weeks) showing increase the expression of UCP1, UCP1 IHC, bar= 40 µm, X40 (**E**). The labeling index of UCP1 was expressed as the percentage of positive area cells in about 8 high power fields using image J analysis software (NIH, USA) (**F**). Values presented are the mean ± SE. Columns with different letters means significant difference (*p* < 0.05) from the other groups.

Figure 5 showed protein expression of UCP1 in brown adipose tissue of control and treated groups. Feeding rats of the HFD group HFD significantly reduced (*p* < 0.05) protein expression of UCP1 in brown adipose tissues compared with the control group fed basal diet (Figure 5C,F). However, administration of rats fed HFD (HFD+ CME and HFD then normal diet+ CME groups) with CME significantly increased (*p* < 0.05) protein expression of UCP1 in brown adipose tissues compared with the HFD fed group (HFD group) (Figure 5D–F). In addition, CME itself significantly increased UCP1 protein expression in brown adipose tissues of the CME group compared with the control group fed basal diet (Figure 5B,F).

## 4. Discussion

Obesity and dyslipidemia are metabolic disorders that represent a big problem on human health that increase the risk of many diseases such as cardiovascular disease, diabetes mellitus and hypertension [1]. However, *Commiphora myrrha* has antioxidant, hypoglycemic, hypolipidemic and anti-diabetic activities [37].

The results of our study revealed that feeding rats HFD increased food intake, liver and body weights and induced hyperglycemia, dyslipidemia, hyperketonemia, hypoleptinemia, hypoadiponectinemia and oxidative stress in hepatic tissue accompanied with alteration of liver structure and function and decreased protein expression of UCP1 in brown adipose tissue. This increase in body weight may attribute to the increase of food intake, which in turn enhanced excess energy and buildup of adiposity because HFD consumption favors more fat storage than fat oxidation in muscle [38]. The other possible cause of HFD induced increased body weights, hyperglycemia and dyslipidemia in the current study is the reduction of leptin, adiponectin and UCP1 protein expression, which play important roles in the regulation of food intake, insulin sensitivity and energy metabolism. Leptin plays an important role in the control of appetite [39] through its action on the hypothalamus to control appetite, food intake, energy metabolism and outflow of the sympathetic nervous system [40]. Hence, impairment of its secretion and/or action results in weight gain by sending an inappropriate signal to the brain, which consequently reduced satiety response. The leptin secretion and circulating leptin level are manipulated by the kind of diet as reduced carbohydrate not fat intake in obese human subjects is associated with lower leptin concentration [41]. In addition, feeding rats HFD for 4 to 14 weeks reduces leptin secretion leading to increased body weight gain [42]. The possible reasons behind HFD reduced leptin serum levels may be HFD decreased insulin-mediated glucose metabolism in adipocytes, which associated with lower leptin expression and secretion from adipocytes [43,44] because HFD induced lipolysis increases the expression of peroxisome proliferator–activated receptor gamma [45], which reduces the expression of leptin in adipose tissue [46]. 

Adiponectin has anti-atherogenic and anti-inflammatory and insulin sensitizing properties and its plasma concentrations are decreased in obese individuals and type 2 diabetic mellitus (DM2) [9,47]. UCP1 is an important gene that controls the development of obesity and DM2 [13]. As it plays a great role in the regulation of cold and diet induced thermogenesis, metabolic and energy balance and decreasing reactive oxygen species (ROS) production, which implicated in the pathogenesis of obesity and/or DM2 [14]. Several studies stated that there was a relation between reduced expression of UCP1 in adipose tissue of obese subjects and the polymorphism of the 3826G allele of UCP1 gene [48], which in turn associated with the obesity or other obesity disorders as DM2 [49]. Therefore, HFD may contribute to weight gain by reducing leptin, adiponectin and UCP1 production.

The dyslipidemic effect of HFD in the current study may due to the consumption of HFD alters lipid profile [38] through increasing fatty acid delivery to the liver that increases TG synthesis accompanied by an increase of VLDL synthesis [50]. Moreover, HFD up regulates lipogenesis pathway [51] leading to increases of the production of triglyceride and total cholesterol, which in turn enhanced insulin resistance and glucose intolerance [52] and consequently hepatomegaly.

The HFD hyperketonemia and hepatic dysfunction may due to consumption of HFD increases serum level of ketone bodies and serum activities of ALT and AST [4]. Obesity and overweight were correlated with a decrease in insulin sensitivity and development of diabetes mellitus type-2, which in turn increase ALT activity [53]. The hyperglycemic and dyslipidemic effects of HFD in the current study may induce oxidative stress and alter the antioxidant enzyme system in obese rats, which represented by elevated level of lipid peroxidation biomarker, MDA, and reduced activity of GR. Glutathione reductase catalyzes the conversion of oxidized glutathione to reduced glutathione (GSH) in the hepatic tissues of obese rats. Reduced glutathione acts as antioxidant, involved in transport of amino acid, and it is a substrate for glutathione peroxidases and glutathione s-transferases, which responsible for organic peroxide detoxification and xenobiotics metabolism respectively [54]. High fat diet induced oxidative stress in our study led to pathological changes in the hepatic tissues of HFD fed rats due to consumption of HFD induces diffuse and extensive microvesicular steatosis with great enlargement of hepatocyte and presence of intracytoplasmic acidophilic globular body [4]. 

On the contrast, administration of rats fed HFD with CME ameliorated HFD increased food intake, body weight gain and livers weights in rats fed. This finding may be attributed to decreased food intake (Figure 2). This decrease in food intake led to low caloric intake, low energy excess and decrease buildup of adiposity. These findings were parallel with those of [21] who demonstrated that administration of obese rats with CM decreases body gain. These decreases in food intake and body weight gain may due to CME normalized serum leptin levels, which control food via its action on the satiety center in the brain and subsequently regulates body weight gain [55]. *Commiphora myrrha* extract normalized serum leptin levels through different possible pathways such as increasing UCP1 expression in brown adipose tissue, which subsequently increased energy expenditure and reduced obesity development which reduced leptin synthesis and secretion by adipocytes [43,44]. In addition, CME increased insulin mediated glucose metabolism, which increased leptin synthesis and secretion by adipocytes [43,44]. *Commiphora myrrha* extract reversed the effect of HFD on UCP1 protein expression in brown adipose tissue may due to its content of retinol (vitamin A), which up regulates UCP1 gene expression in brown adipose tissue. [56]. This explanation can be confirmed by the finding of [57] who indicated that feeding obese rats’ vitamin A reduces body weight gain, adiposity index, and retroperitoneal white adipose tissue mass. In addition, guggulsterone (Myrrha sterols) acts as an anti-obesity agent as it decreases adipogenesis through preventing the differentiation of preadipocytes into mature adipocytes [58] and decreases food intake and body weight gain in rats fed HFD due to it reduces the plasma ghrelin, glucose, triglyceride levels and increased plasma leptin, serotonin, dopamine levels [59]. Moreover, it up-regulates the expression of a thermogenic marker, UCP1, in mature adipocytes. The hypocholestermic effect of CME may be related to the inhibition of cholesterol synthesis via antagonism of foresenoid X receptor and bile receptor [60] as Guggulsterone is structurally similar to bile acids [61] and through its effect on UCP1 expression. Thus, Guggulsterone acts as anti-obesity agent via reducing adipogenesis, increasing lipolysis in white adipose tissue and inducing trans-differentiation of white adipocytes into beige that decrease body weight. Furthermore, Ref. [62] indicated that guggulsterone enhances mitochondrial density, biogenesis of peroxisome proliferator-activated receptor gamma coactivator 1-alpha (PGC1α) and peroxisome proliferator-activated receptor-γ (PPARγ), and increased expression beige adipocyte phenotype markers, UCP1, t-box transcription factor 1 (TBX1), and β-3 adrenergic receptor (β-3AR). The anti-obesity effect of adiponectin may be attributed to the activation of AMPK increasing fatty acid oxidation and reduction of serum glucose [12,63]. From the above mentioned findings, it has become clear that the reduced protein expression of leptin, adiponectin and UCP1 is one the most of important possible causes that increased body weight; hyperglycemia and dyslipidemia in HFD fed rats that were restored by administration of HFD fed rats with CME. The hypoglycemic effect of CME in our study confirmed the findings of [18,19] that indicated the administration of myrrh extract decreases the fasting blood glucose in diabetic rats. This hypoglycemic effect of CME may be attributed to the presence of germacrene B and benzoquinone compounds in CME (Table 3) as [64] stated that administration of rats with Embelin, 1,4-benzoquinone, lowered blood glucose and serum insulin levels. In addition, CME contains anti-hyperglycemic compounds, furanoeudesma-1,3-diene and 2-O-acetyl-8,12-epoxygermacra-1 [37] and has stimulatory effect on β cells division and insulin secretion [18,65]. Benzofuranebisindole hybrids have been shown to have in vitro antioxidant effect and in vivo anti-dyslipidemic activity as it decreases total cholesterol, triglycerides and phospholipids through increasing the plasma lecithin cholesterol acyltransferase activity, which plays a key role in lipoprotein metabolism increasing HDL-C in serum level [66].

The demonstrated hypolipidemic effect of CME in our study confirmed the findings of the previous studies, which found that administration of rats fed high fructose diet with Commiphora Mukul decreases lipid profiles and the atherogenic index [19] and Commiphora Molmol extract decreases lipid profile and atherogenic index in obese rats [21] The hypocholesteremic effect might due to the presence of hexadecanoic acid, 9 and 12-octadecenoic acids in CME (Table 3), which have been proven to have inhibit the activity of pancreatic lipase suppressing lipid digestion and thereby diminishing entry of lipids into the body [67]. In addition, CME contains germacrene B, sesquiterpene (Table 3), which have a hypolipidemic effect [68]. 

The other possible reason behind the anti-obesity effect of CME in our study is its antioxidant activity as our results revealed that administration of rats fed HFD with CME alleviated HFD induced oxidative stress through decreasing lipid peroxidation and enhancing the antioxidant enzyme activity of hepatic tissues (Table 6) and it has been reported that administration of high fructose diet fed rats with Commiphora Mukul decreases MDA levels while increases GR activity that preserving GSH contents in hepatic tissues [19]. These results may be attributed to the presence of certain bioactive compounds in CME that have antioxidant effect such as 11, 14-eicosadienoic acid, 9, 12-octadecenoic acid methy ester, germacrene B and isochiapin B (Table 3). These findings were in line with [69] as he found that *Centaurea centaurium* L. methanolic root extract has potent antioxidant properties due to the presence fatty acids: 11,14-eicosadienoic acid methyl ester, 9-octadecenoic acid and terpenes such as β-bisabolene, β and [70] indicated that Juniperus phoenicea has powerful anti-diabetic, anti-obesity and antioxidant activities due to it contains Germacrene B and other antioxidants. Moreover, Ref. [71] showed that elemene administration to atherosclerotic rabbit decreases obesity via reduction of the infiltration of macrophage, also reduction of the levels of TC, TG, and LDL-C and the inflammatory factors as TNF-Į and IL-6 levels in vitro. Ref. [72] showed that the inhibitory effect of elemene on atherosclerosis mediated by reduction of lipid peroxidation, enhancement of anti-oxidant defense system, and suppression of inflammatory chemokine expression in atherosclerotic mice induced by HFD for 16 weeks. In addition, 1-heptatriacotanol is one of the most effective components in CM, as [73] reported that Basella alba leaf extract, which contain1-heptatriacotanol has hypocholesterolemic effect through inhibition of HMG-CoA reductase enzyme. These antioxidant activities of CME protected hepatic tissues of HFD fed rats (Figure 4). That antioxidant property of CME ameliorated the detrimental effects of HFD on the liver structure and function as it has been found that treatment of rats fed high fructose diet with CM improves liver tissues structure and induces regeneration of hepatocyte around central vein [20]. The protective effects of CME on hepatocytes may be related to its phytochemical constituents, sesquiterpene, reynosin, bisabolene, elemene and curezerine (benzofuran) compounds in CME, which have antioxidant and hepatoprotective effects as [20] indicated the presence of curezerine, delta-elemene, beta-elemene, 3-[(E)-2-phenyl-1-propenyl] cyc, bicycle [3.1.1] hept-2-ene-2-car, 8-isopropenyl-9-isopropyltetra, and 2,4-bis (3-methyl-1-pentynyl)-4 in CME. These compounds were demonstrated to have cytoprotective, antioxidant and anticancer effects [74,75]. The protective effects of CME on hepatic tissue improved liver function as our studies and [18,20] demonstrated that treatment of animals with CME reduced serum activities of AST and ALT. These results may due to the presence of reynosin compounds in CME (Table 3) as reynosin has been shown to inhibit thioacetamide induced apoptosis and hepatocellular DNA damage through decreasing mRNA expression of proapoptotic Bax and increasing mRNA expression of antiapoptotic Bcl-2, Bcl-XL in primary rat hepatocytedecreases and reduced the activities of serum AST and ALT in thioacetamide intoxicated mice [75]. Taken together from the aforementioned discussion it can be postulated that CME reduced HFD induced dyslipidemia and increased body weight gain through different pathways such as up-regulation of leptin, UCP1 and adiponectin expression and antioxidant activity. 

## 5. Conclusions

This study indicated that HFD increased food intake and body weight gain and induced dyslipidemia and hyperglycemia in rats through reducing leptin, adiponectin and UCP1 protein production. However, CME ameliorated HFD increased food intake, body weight gain, hyperglycemia and dyslipidemia through normalization of HFD reduced leptin, adiponectin and UCP1 synthesis and production as well as its antioxidant activity suggesting that CME is a potent anti-obesity agent.

## Figures and Tables

**Table 1:** Chemical composition of basal diet.

Basal Diet Chemical Composition	(g %)
**Protein**	17%
**Fat**	4.9%
**Choline chloride**	2%
**Vitamin mixture**	1%
**Salt mixture**	3.5%
**Fiber**	3.44%
**Carbohydrates**	68.16%

**Table 2:** Chemical composition of high fat diet.

High Fat Diet Chemical Composition	(g %)
**Crude protein**	16.68%
**Fat**	31.59%
**Carbohydrates**	51.73%
**Energy**	4.66 kcal/g

**Table 3:** Identified compounds from *Commiphora myrrha* resin extract.

Compound Name	RT/min	Area %	MW	MF
Benzofuran	19.38	8.09	216	C15H20O
Germacerene B	20.78	0.71	204	C15H24
ç-Elemen	20.78	0.71	204	C15H24
1,4-Benzoquinone	25.99	0.68	312	C21H28O2
Isochiapin B	27.09	1.15	346	C19H22O6
Bisabolene epixod	28.6	1.37	220	C15H24O
Hexadecanoic acid, methyl ester	28.89	3.82	270	C17H34O2
Retinol	30.4	3.12	286	C20H30O
Reynosin	30.72	1.21	248	C15H20O3
9,12-Octadecadienoic acid (Z,Z)-, methyl ester	32.10	5.96	294	C19H34O2
11-Octadecenoic acid, methyl ester	32.26	12.67	296	C19H36O2
11,14-Eicosadienoic acid, methyl ester	33.02	0.21	322	C21H38O2
1-Heptatriacotanol	34.00	0.51	396	C27H56O

Retention time, RT; Molecular weight, MW; Molecular formula, MF.

**Table 4:** Effects of HFD and/or *Commiphora myrrha* resin extract on blood glucose level and serum lipid profile of rats after 8 weeks.

Groups Parameters	Basal Diet		HFD	
	Control	CME	Control	C ME
**Glucose (mg/dL)**	101.50 ± 1.04^c^	95.40 ± 3.44^c^	154.14 ± 1.32^a^	122.14 ± 2.46^b^
**TC (mg/dL)**	208.17 ± 4.91^bc^	186.5 ± 13.37^c^	232.70 ± 1.42^a^	213.00 ± 2.13^b^
**TAG (mg/dL)**	147.33 ± 11.09^c^	132.12 ± 6.91^c^	197.67 ± 0.67^a^	177.33 ± 2.49^b^
**HDL (mg/dL)**	56.50 ± 5.43^a^	56.75 ± 1.33^a^	40.60 ± 1.16^c^	47.11 ± 1.45^b^
**LDL (mg/dL)**	122.20 ± 5.07^bc^	103.3 ± 11.37^c^	153.33 ± 1.38^a^	129.69 ± 1.39^b^
**VLDL (mg/dL)**	29.46 ± 2.21^c^	26.42 ± 1.38^c^	39.15 ± 0.21^a^	35.46 ± 0.50^b^

Values are expressed as means ± SE (standard errors). The mean difference is significant at *p* < 0.05. Values with different letters in the same ROW were significantly different. High fat diet, HFD; *Commiphora myrrha* resin extract, CME; total cholesterol, TC; Triacylglycerol, TAG; High density lipoprotein, HDL; Low-density lipoprotein cholesterol, LDL, Very low density lipoprotein cholesterol, VLDL.

**Table 5:** Effect of HFD and/or *Commiphora myrrha* resin extract on blood glucose level, serum levels of lipid profile, ketone bodies and activities liver function biomarkers of rats after 14 weeks.

Groups Parameter	Basal Diet		HFD		(14 Weeks)	(8 Weeks) Then
	Control	CME	Control	CME	Normal Diet & CME (6 Weeks)	
**Glucose (mg/dL)**	115.25 ± 2.74^c^	75.13 ± 3.37^d^	159.10 ± 8.88^a^	137.44 ± 2.01^b^	88.50 ± 3.05^d^	
**TC (mg/dL)**	171.25 ± 7.51^d^	153.25 ± 7.10^d^	242.50 ± 7.50^a^	218.89 ± 6.65^b^	193.60 ± 3.19^c^	
**TAG (mg/dL)**	108.25 ± 7.30^d^	92.63 ± 7.19^e^	195.30 ± 1.11^a^	162.56 ± 5.46^b^	147.80 ± 2.87^c^	
**HDL (mg/dL)**	57.87 ± 2.34^a^	54.12 ± 2.28^a^	26.40 ± 0.67^c^	33.66 ± 2.33^b^	34.00 ± 0.57^b^	
**LDL (mg/dL)**	84.47 ± 11.34^d^	80.37 ± 7.48^d^	177.14 ± 7.30^a^	148.27 ± 6.40^b^	126.44 ± 3.26^c^	
**VLDL (mg/dL)**	21.65 ± 1.46^d^	18.52 ± 1.43^e^	39.06 ± 0.22^a^	32.51 ± 1.09	29.56 ± 0.57^c^	
**Ketone bodies**	0.94 ± 0.03^c^	0.79 ± 0.01^c^	2.57 ± 0.09^a^	1.24 ± 0.06^b^	1.13 ± 0.03^b^	
**ALT (U/L)**	22.37 ± 1.60^c^	24.13 ± 1.46^c^	43.5 ± 0.93^a^	38.87 ± 0.74^b^	39.40 ± 0.67^b^	
**AST (U/L)**	25.50 ± 1.49^c^	26.00 ± 1.45^c^	45.62 ± 0.49^a^	39.12 ± 0.58^b^	40.88 ± 0.48^b^	

Values are expressed as means ± SE; The mean difference is significant at *p* < 0.05. Values carrying different letters in the same ROW were significant differences. High fat diet, HFD; *Commiphora myrrha* resin extract, CME; total cholesterol, TC; Triacylglycerol, TAG; High density lipoprotein, HDL; low density lipoprotein, LDL; very low density lipoprotein, VLDL; ketone bodies, alanine aminotransferase, ALT; aspartate aminotransferase, AST.

**Table 6:** Effect of HFD and/or *Commiphora myrrha* resin extract on hepatic tissue content of malondialdehyde and activity of glutathione reductase after 14 weeks.

	Normal Diet		HFD		
			(14 Weeks)		(8 Weeks) Then
	Control	CME	Control	CME	Normal Diet & CME (6 Weeks)
**MDA (nmol/g)**	11.24 ± 0.58^c^	11.94 ± 0.9^c^	23.46 ± 1.07^a^	17.20 ± 0.54^b^	16.57 ± 0.7^b^
**GR (U/L)**	9.65 ± 0.98^a^	8.84 ± 0.80^a^	2.41 ± 0.98^c^	5.62 ± 0.98^b^	5.62 ± 0.98^b^

Values are expressed as means ± SE. The mean difference is significant at *p* < 0.05. Values carrying different letters in the same ROW were significant differences. High fat diet, HFD; *Commiphora myrrha* resin extract, CME; malondialdehyde, MDA; glutathione reductase, GR.

**Table 7:** Effect of HFD and/or *Commiphora myrrha* resin extract on Serum levels of leptin and adiponectin of rats after 14 weeks.

Groups Parameter	Normal Diet		HFD		
			(14 Weeks)	(8 Weeks) Then	
	Control	CME	Control	CME	Normal Diet + CME (6 Weeks)
**Leptin (pg/mL)**	315.89 ± 45.69^a^	304.33 ± 24.27^a^	169.08 ± 17.9^b^	362.25 ± 65.1^a^	365.4 ± 29.7^a^
**Adiponectin (mg/L)**	18.22 ± 1.69^a^	14.15 ± 1.11^b^	9.15 ± 2.85^c^	17.39 ± 0.94^ab^	19.60 ± 0.94

Values are expressed as means ± SE. The mean difference is significant at *p* < 0.05. Values carrying different letters in the same ROW were significantly different. High fat diet, HFD; *Commiphora myrrha* resin extract, CME.
